# Effect of the genetic mutant G71R in uridine diphosphate-glucuronosyltransferase 1A1 on the conjugation of bilirubin

**DOI:** 10.1515/biol-2022-0021

**Published:** 2022-03-18

**Authors:** Hong Chen, Danni Zhong, Zongyan Gao, Xiaojing Wu

**Affiliations:** Department of Pediatrics, The First Affiliated Hospital of Guangxi Medical University, No. 6 Shuangyong Road, Nanning 530021, Guangxi, China

**Keywords:** lentiviral vector, G71R mutant, hyperbilirubinemia, UDP-glucuronosyltransferase 1A1

## Abstract

We aimed to investigate the effect of the genetic mutant G71R (c. 211G > A) in uridine diphosphate (UDP)-glucuronosyltransferase 1A1 (UGT1A1) on the glucuronidation of unconjugated bilirubin. The UGT1A1 wild-type and mutant G71R gene sequences were inserted into the lentiviral vector GV358 plasmid and then transfected into COS-7 cells. Real-time polymerase chain reaction and western blot analyses were used to determine mRNA and protein expression levels of UGT1A1, respectively. High-performance liquid chromatography was used to quantitate the levels of conjugated bilirubin. The results showed no significant difference in the mRNA and protein expression levels between the UGT1A1 wild-type and G71R homozygous and heterozygous mutants. The level of conjugated bilirubin reached a maximum in wild-type UGT1A1-transfected COS-7 cells. However, relative to the UGT1A1 wild-type, conjugated bilirubin concentrations were 71 and 22% with G71R heterozygous- and G71R homozygous-transfected COS-7 cells, respectively. In conclusion, we successfully established *in vitro* cell models of the UGT1A1 wild-type and the G71R homozygous and heterozygous mutants using a lentiviral vector. Furthermore, the catalytic activity for unconjugated bilirubin was lower in the mutant G71R than the UGT1A1 wild-type enzyme, and a weaker effect was observed in the homozygote.

## 1 Introduction

Neonatal hyperbilirubinemia is one of the most common clinical conditions in the neonatal period, accounting for 49.1% of all hospitalized neonatal cases [1]. It is usually transient, but severe cases can lead to bilirubin encephalopathy (and if chronic to kernicterus), ultimately affecting hearing, motion, intelligence, and even causing death. Severe hyperbilirubinemia occurs in 8–10% of newborns [2]. Neonatal hyperbilirubinemia has complex pathogenesis, and there are several risk factors, including ABO incompatibility, infection, low birth weight, and glucose-6-phosphate dehydrogenase deficiency. A large number of studies have shown that uridine diphosphate (UDP)-glucuronosyltransferase 1A1 (UGT1A1) gene mutations are one of the main genetic causes of hereditary unconjugated hyperbilirubinemia [3,4,5,6].

UGT1A1 is a critical enzyme in bilirubin conjugation, encoded by *UGT1A1* [7]. *UGT1A1* is located on human chromosome 2 (region 2q37) and comprises a promoter TATA box and exons 1–5. Mutations in *UGT1A1* cause the reduction of bilirubin conjugation, leading to hereditary unconjugated hyperbilirubinemia. More than 130 types of *UGT1A1* mutations have been discovered thus far, including promoter and coding region mutations, with racial and regional differences [8]. Among these mutations, G71R is the most common, where the codon changes from G to A at nucleotide 211, causing glycine to be replaced by arginine at position 71 in the corresponding protein product. Several studies have demonstrated that G71R is a risk factor for neonatal hyperbilirubinemia in the Asian population [9,10,11,12]. In studies performed in China, the high allele frequency of the G71R mutation was responsible for neonatal hyperbilirubinemia [13,14]. Furthermore, several other studies have found that the G71R mutation is not only related to prolonged unconjugated hyperbilirubinemia [15] but is also a major cause of breast milk jaundice [16,17]. In addition, other studies have shown that the G71R mutation was the most common cause of Gilbert’s syndrome (GS) and Crigler–Najjar syndrome type II (CN-II) [5,18].

According to previous studies, the allele gene frequency of G71R in neonatal hyperbilirubinemia was higher than that in normal neonates in Guangxi, China. Compared with normal neonates, homozygous neonates with G71R mutation had higher total serum bilirubin concentrations 72 h after birth and a higher possibility of developing bilirubin encephalopathy. The researchers concluded that in Guangxi, China, the G71R mutation was closely associated with neonatal hyperbilirubinemia [13,19]. Furthermore, other studies have shown lower UGT1A1 enzyme activity in infants with homozygous G71R mutation than in infants with heterozygous G71R mutation [20,21]. Therefore, the authors speculated that different expression levels of UGT1A1 in G71R mutation and UGT1A1 wild-type might be the mechanism underlying the development of neonatal hyperbilirubinemia.

## 2 Materials and methods

### 2.1 Cell culture

The COS-7 cell line (derived from African green monkey renal fibroblasts and transformed by the SV40 viral gene) was purchased from the Shanghai Cell Bank of the Chinese Academy of Sciences, China. The cells were incubated in a 37°C humidified incubator containing 5% CO_2_ and Dulbecco’s minimum essential medium (DMEM) (Gibco, NY, USA) supplemented with 5% fetal bovine serum (FBS, Gibco, NY, USA), 100 U/mL penicillin G, and 100 µg/mL streptomycin (Gibco, NY, USA).

### 2.2 Amplification of the target gene

Polymerase chain reaction (PCR) primers were designed based on the coding region fragment DNA (cDNA) of *UGT1A1* in GenBank (GenBank accession number: NM_000463). The *UGT1A1* PCR primer pair was as follows: 5′-GAGGATCCCCGGGTACCGGTCGCCACCATGGCTGTGGAGTCCCAGGGCGGACGCCCAC-3′/5′-TCCTTGTAGTCCATACCATGGGTCTTGGATTTGTGGGCTT TC-3′. Then, the cDNA of *UGT1A1* was inserted into the GV358 vector. The mutation was introduced using an In-Fusion PCR Cloning Kit (Clontech, San Francisco, USA) following the manufacturer’s protocols. The primer sets used to introduce mutations were as follows (the mutation point is underlined): 5′-GTACATCAGAGACAGAGCATTTTACACCTTGAAG-3′/5′-GTAAAATGCTCTGT CTCTGATGTACAACGAGG-3′ were used for G71R (211G > A). The PCR conditions were as follows: denaturation at 98°C for 5 min, denaturation at 98°C for 10 s, annealing at 55°C for 10 s, extension at 72°C for 90 s, and a final extension at 72°C for 8 min. The total PCR process comprised 30 cycles. Sequencing confirmed the mutation site and other parts of the *UGT1A1* cDNA.

### 2.3 Transfection

Approximately 8 × 10^5^ COS-7 cells were seeded into each well of a six-well plate and cultured in DMEM supplemented with 10% FBS for 24 h before transfection. Negative control lentiviral vectors and lentiviral vectors overexpressing *UGT1A1* and containing G71R mutation were constructed by GeneChem (Shanghai, China). COS-7 cells were transfected with the appropriate lentiviral vectors following the manufacturer’s protocols. The following expression models were created: normal *UGT1A1* (wild-type), homozygote for G71R, heterozygote for G71R, mock-transfected cells, and untransfected COS-7 cells. In the heterozygous model of G71R, the GV358 vector with normal *UGT1A1* cDNA and the GV358 vector with the mutated G71R-*UGT1A1* cDNA were cotransfected (the wild-type vector:mutant vector ratio was 1:1) into the cells. The concentration of the lentiviral vector was 1 × 10^8^ TU/mL. Hexadimethrine bromide (polybrene, a final concentration of 50 μg/mL) was added to enhance the lentiviral infection ratio. A culture medium containing 1 μg/mL puromycin was added, and a stable COS-7 cell line was selected 72 h after transfection. Green fluorescence was observed under a fluorescence microscope (Olympus, Tokyo, Japan), and an FC500/MPL flow cytometer (Beckman Coulter, CA, USA) was used to determine the infection efficiency.

### 2.4 DNA analysis

Briefly, transfected cells were washed twice with cold phosphate-buffered saline, digested, and centrifuged. DNA was extracted using a TIANamp Genomic DNA Kit DP304 (TIANGEN, Beijing, China). The primer was designed according to the mutation point. The forward primer was 5′-TGCTGGGAAGATACTGTTGAT-3′, and the reverse primer was 5′-GCCAGACAAAAGCATAGCAGA-3′. A PCR mixture (50 µL) was prepared to contain 2 µL of forward and reverse primers, 20 µL of ddH_2_O, 25 µL of 2× Taq Mastermix (CWBio, Beijing, China), and 1 µL of the DNA template. The PCR conditions were as follows: predenaturation at 94°C for 2 min, denaturation at 98°C for 30 s, annealing at 60°C for 30 s, extension at 72°C for 30 s, and extension at 72°C for 2 min. The total PCR process comprised 30 cycles, and the PCR products were separated by 2% agarose gel electrophoresis and sequenced by Beijing Genomics Institute (Beijing, China).

### 2.5 Quantitative real-time fluorescent PCR

Total RNA was extracted using TRIzol (Invitrogen, Carlsbad, USA). A total of 2 μg of RNA was reverse transcribed into cDNA using a Revertaid First Strand cDNA Synthesis Kit (Invitrogen). The primers used for detecting *UGT1A1* and *β-actin* by qRT-PCR were as follows: 5′-TAGTTGTCCTAGCACCTGACGC-3′/5′-TCTTTCACATCCTCCCTTT GG-3′*(UGT1A1*) and 5′ACTCCATCATGAAGTGTGACG-3′/5′-CATACTCCTGCTT GCTGATCC-3′ (*β-actin*). *UGT1A1* was amplified with an SYBR Green PCR Master Mix Kit (Roche, Indianapolis, USA) using an ABI 7500 qRT-PCR system (Applied Biosystems, Foster, USA). The reaction conditions were as follows: a predenaturation step at 95°C for 10 min, followed by 40 cycles of the denaturation step at 95°C for 15 s, and annealing and extension at 60°C for 1 min. The relative mRNA expression was calculated using the 2^−ΔΔCt^ method and normalized to *β-actin* [22].

### 2.6 Western blot analysis

Total protein of COS-7 cells was isolated using radioimmunoprecipitation assay lysis buffer (Solarbio, Beijing, China) containing the protease inhibitor phenylmethanesulfonyl fluoride (Solarbio). The protein concentration was assessed using a bicinchoninic acid (BCA) protein assay reagent kit (Vazyme, Nanjing, China). Equal amounts of total protein (30 μg) were added to the wells of 10% sodium dodecyl sulfate-polyacrylamide gel electrophoresis and separated. The proteins were transferred to polyvinylidene fluoride membranes (Millipore, Boston, USA). Subsequently, the membrane was blocked with 5% nonfat milk at room temperature for 1 h. Then, the membranes were incubated with primary antibodies against UGT1A1 and β-actin (1:1,000, Abcam, Cambridge, UK) for 12–15 h at 4°C, washed with tris-buffered saline Tween (TBST) three times, and incubated with horseradish peroxidase-conjugated anti-rabbit immunoglobulin G fluorescent secondary antibody (1:8,000, Abcam) at room temperature for 2 h. Fluorescence bands corresponding to UGT1A1 and β-actin were imaged using an Odyssey Fc scanner (LI-COR, NE, USA). The grayscale values of UGT1A1 and β-actin proteins were measured using LI-COR Odyssey 3.0 analytical software; the relative protein expression level is equal to the grayscale value of UGT1A1 protein/β-actin internal reference protein.

### 2.7 UGT1A1 activity

Bilirubin and its glucuronides were separated on a high-performance liquid chromatography (HPLC) column (reverse-phase Diamonsil C18 column, Dikma, Beijing, China) using a Shimadzu LC-20A HPLC system (Kyoto, Japan), and the system control and data analyses were carried out using a Shimadzu LC solution workstation (Shimadzu). The chromatographic conditions and incubation procedure for bilirubin glucuronidation were as previously described [23]. Ultrasound was used for protein extraction, and a BCA protein assay reagent kit (Vazyme) was used to assess the protein concentration. An equal amount of protein (50 μg) was added during the incubation procedure for bilirubin glucuronidation. Standard samples were prepared for calibration curves, and the final concentrations of bilirubin in the standard samples were 0.25, 0.5, 0.75, 1.0, 1.25, 1.5, and 2 μM. The following control groups were designed: a control group containing bilirubin without uridine diphosphoglucuronic acid (UDPGA) in the reaction system and another group containing UDPGA but no bilirubin.

### 2.8 Statistical analyses

The statistical analysis was carried out using SPSS 16.0 (SPSS Inc., IL, USA). Each parameter is presented as the mean ± standard deviation. The normally distributed data were analyzed by one-way analysis of variance with a *post hoc* test of the least significant difference. The nonnormally distributed data were analyzed using the Kruskal–Wallis test with the *post hoc* Mann–Whitney test. A *P*-value <0.05 indicated a statistically significant difference.

## 3 Results

### 3.1 Wild-type and mutant UGT1A1 were successfully established in COS-7 cells

**Figure 1:**
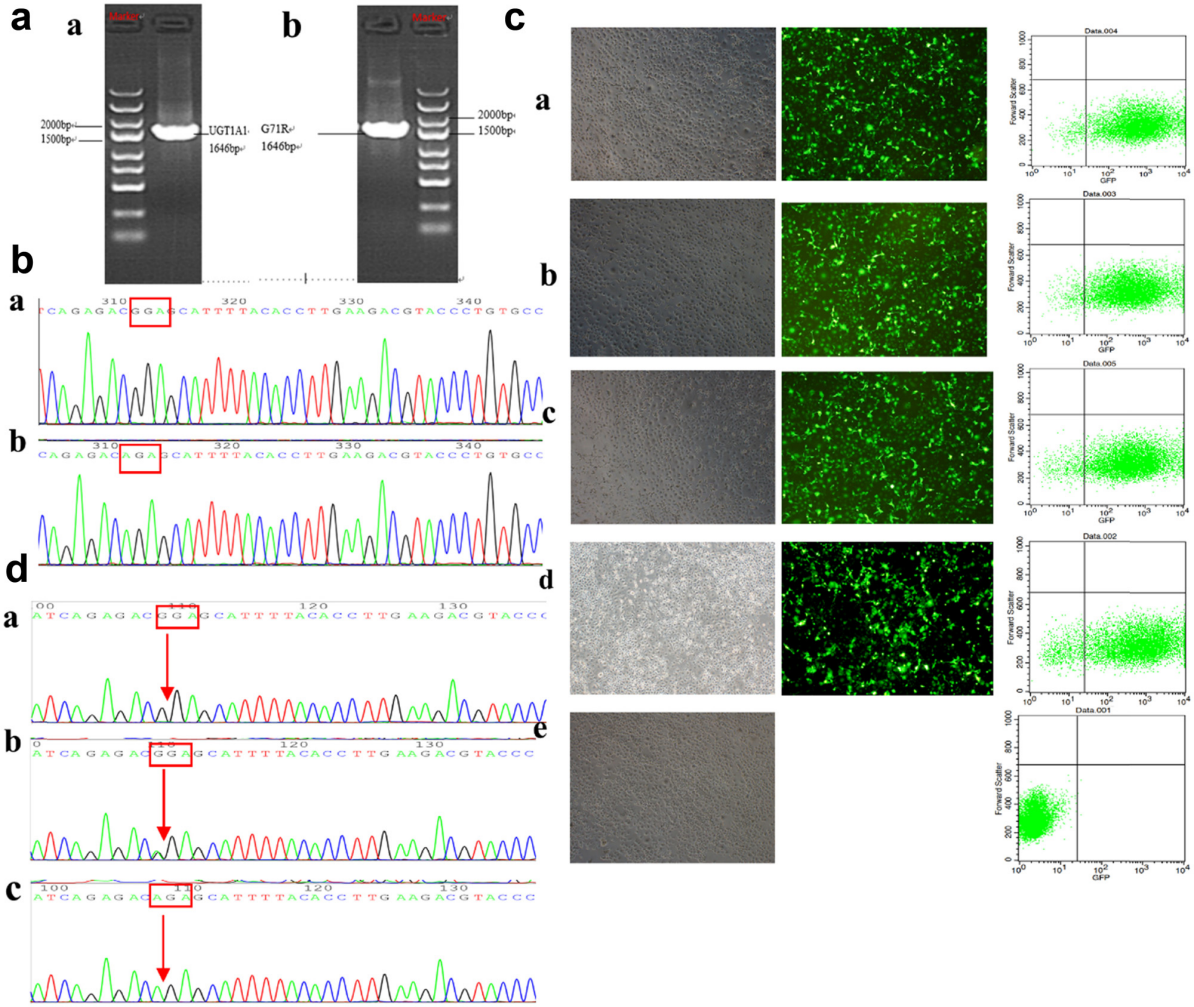
Expression and identification of the wild-type and mutant G71R–UGT1A1. (a) Agarose gel band at 1646 bp derived from the product of PCR amplification. (b(a)) Sequence of the UGT1A1–GV358 recombinant lentiviral vector. (b(b)) Sequence of the G71R–GV358 recombinant lentiviral vector. (c) Expression of green fluorescent protein (GFP) 72 h after transfecting the *UGT1A1* recombinant lentiviral plasmid in COS-7 cells (×200). Flow cytometry detection transfection efficiency. (c(a–d)) Transfection rate of the experimental and empty vector groups was >90%. (c(e)) Transfection rate of the untransfected COS-7 cells was 0.03%. (d) Partial sequencing map of each group. (d(a)) UGT1A1 wild-type; (d(b)) G71R heterozygous mutant (211G > A); and (d(c)) G71R homozygous mutant (211G > A).

The cDNA fragment of *UGT1A1* was amplified by PCR and subcloned into a lentiviral vector. A 1646-bp band was visualized using 1.5% agarose gel electrophoresis (Figure 1a). By sequencing the positive clones, it was found that the subcloned cDNA of *UGT1A1* was in accordance with that of *UGT1A1* released by GenBank. The G71R mutant was consistent with the theoretical results (Figure 1b), indicating that *UGT1A1* was successfully inserted into the GV358 plasmid. Green fluorescence was observed in the experimental group by fluorescence microscopy examination and flow cytometry, and the transfection rate was observed to be >90% (Figure 1c). The DNA fragment of each group was amplified by PCR; then, sequencing analysis indicated that the sequence of the *UGT1A1* wild-type group was the same as the theoretically obtained sequence. The G71R homozygous mutant at the 211th base sequence was homozygous (211G > A), and the G71R heterozygous mutant at the 211th base sequence was heterozygous (211G > A), as shown in Figure 1d. These findings suggested that wild-type and UGT1A1 mutations were successfully established in COS-7 cells.

### 3.2 Mutation of G71R did not affect *UGT1A1* transcription

**Figure 2:**
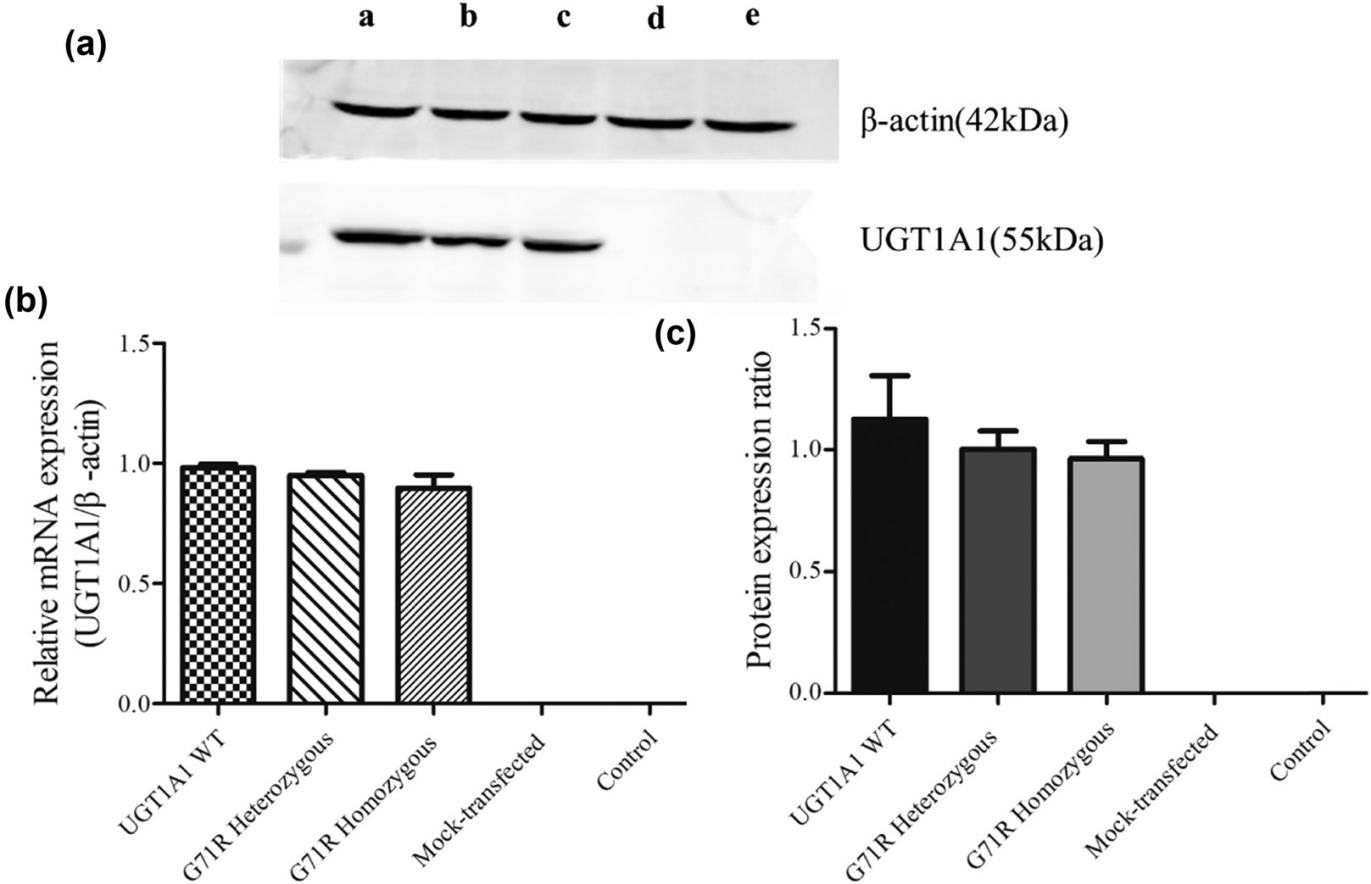
mRNA and protein expression of UGT1A1 were determined using qRT-PCR and western blots, respectively. (a) Western blotting of expressed UGT1A1. (a(a)) UGT1A1 wild-type, (a(b)) homozygote for G71R, (a(c)) heterozygote for G71R, (a(d)) mock-transfected cells, and (a(e)) no transfection COS-7 cells. (b) Relative mRNA expression of UGT1A1. (c)Protein expression ratio of UGT1A1.

To confirm the effects of the G71R mutation, a stable cell line was constructed in which *UGT1A1* was successfully expressed. The mRNA expression of *UGT1A1* in the transfected COS-7 cells was detected, and the expression was found to be higher than that in controls. Western blot analysis revealed a 55 kDa protein band in the wild-type and G71R mutant models but not in the mock transfection model and controls (Figure 2a). No significant difference was found in the mRNA and protein expression levels between the UGT1A1 wild-type and G71R homozygous and heterozygous mutants (Figure 2b and c). This suggested that the mutation of G71R did not affect *UGT1A1* transcription.

### 3.3 Activity of the mutant G71R enzyme on unconjugated bilirubin was lower than that of the UGT1A1 wild-type enzyme

**Figure 3:**
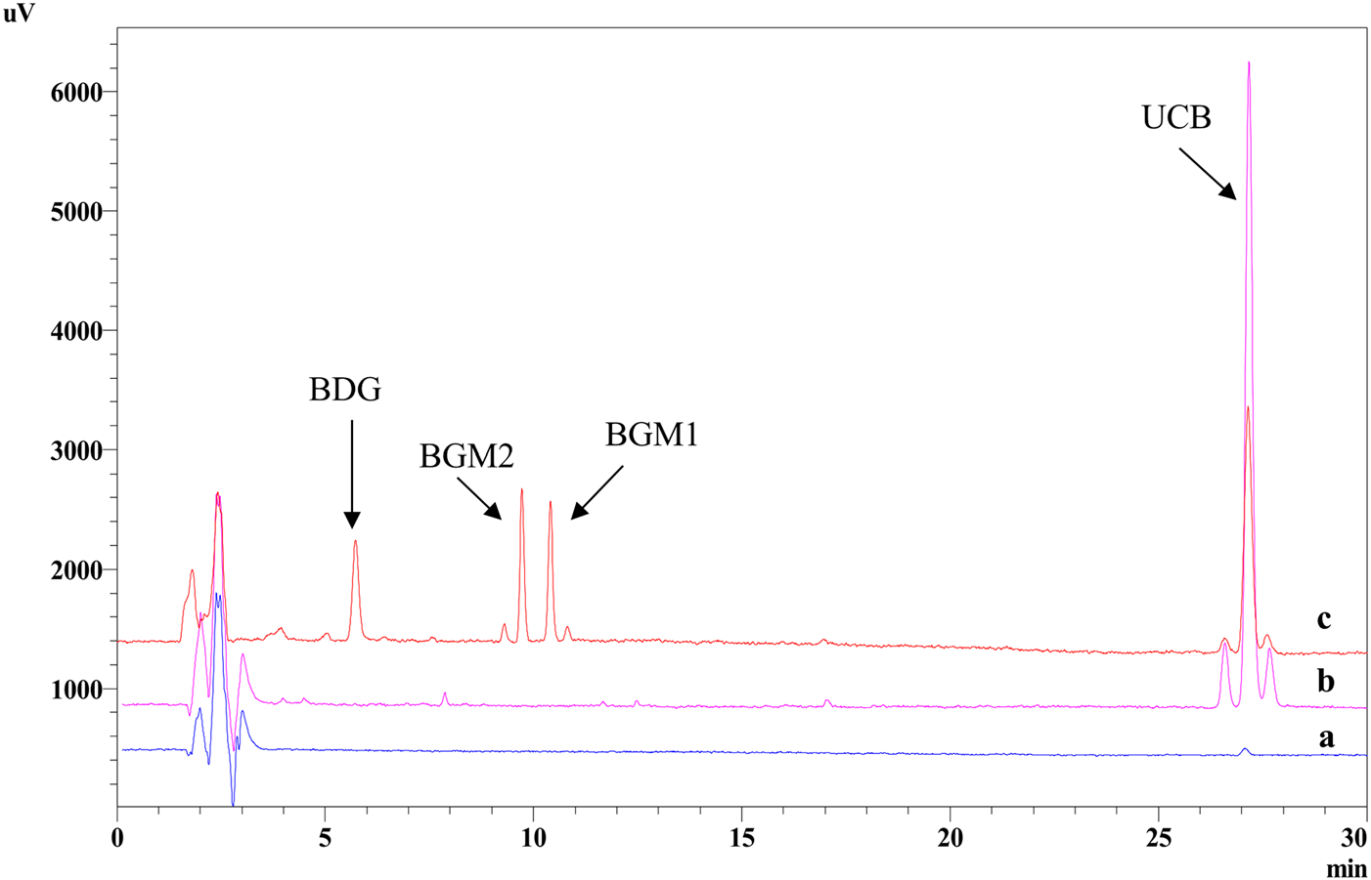
Representative chromatogram for bilirubin glucuronidation by UGT1A1. (a) Control group containing bilirubin without UDPGA in the reaction system. (b) Other control group containing UDPGA but no bilirubin. (c) Bilirubin glucuronidation samples (containing bilirubin and UDPGA).

The representative chromatograms for bilirubin glucuronidation by UGT1A1 are shown in Figure 3. Standard samples were prepared for calibration curves. The combined peak area of bilirubin (the sum of peak areas of bilirubin IX, XIII, and III isomers) was used as the vertical (*Y*) axis, and the concentration of unconjugated bilirubin was used as the horizontal (*X*) axis. Linear regression showed good linearities in the range of 0.25–2 μM bilirubin. The regression equation was *y* = 30,615*x* + 12,883, with a correlation coefficient (*r* ^2^) of 0.998.

**Figure 4:**
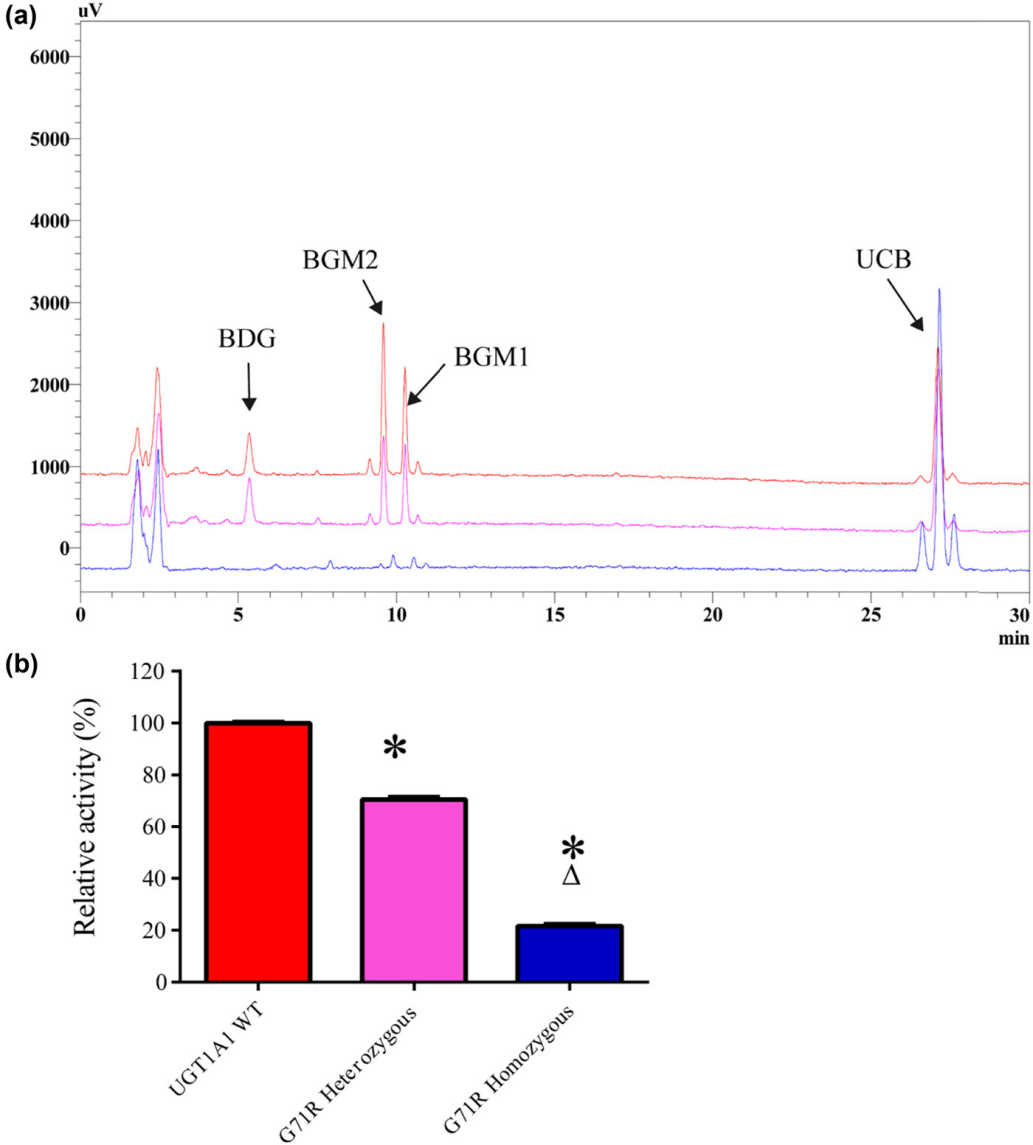
UGT1A1 activity assay. (a) Chromatograms for bilirubin glucuronidation in WT-UGT1A1 and heterozygous and homozygous mutant G71R–UGT1A1 incubation systems. (b) Heterozygous and homozygous mutant G71R–UGT1A1 had 71 and 22% wild-type enzyme activities, respectively. ^*^ *P* < 0.05, compared with the UGT1A1 wild-type group; Δ*P* < 0.05, compared with the heterozygous G71R group.

The increase in the concentration of conjugated bilirubinwas the maximum, which was catalyzed by the UGT1A1 wild-type enzyme. However, the concentration of the conjugated bilirubin decreased in the G71R–UGT1A1 catalytic reaction. The heterozygous and homozygous mutant G71R–UGT1A1 had 71 and 22% wild-type enzyme activity, respectively (Figure 4).

## 4 Discussion

*UGT1A1* mutations are the main pathogeneses of congenital hyperbilirubinemia, with G71R being the most common. Many studies have been trying to identify the pathogenesis of these polymorphisms by analyzing the expression and activity of UGT1A1. In this study, the G71R mutant was successfully constructed by genetic engineering. No significant differences were found in the mRNA and protein expression levels between the *UGT1A1* wild-type and G71R homozygous and heterozygous mutations. However, compared with that of UGT1A1 wild-type enzyme, conjugated bilirubin concentrations were 71 and 22% in the G71R hetero- and homozygous-transfected cells, respectively. This study confirmed that the activity of the mutant G71R enzyme was lower than that of the UGT1A1 wild-type enzyme, and a weaker effect was observed in the homozygote than in the heterozygote.

This study demonstrated that the homozygous and heterozygous G71R mutations had no significant differences in the mRNA and protein expression levels compared with the wild-type *UGT1A1*. This suggested that the G71R mutation did not affect the transcription of *UGT1A1*. However, Jinno et al. constructed *in vitro* cell models of the *UGT1A1* wild-type and the G71R homozygous mutant using plasmid pcDNA3.1 and COS-1 cells. These authors found that the protein expression of UGT1A1 in the G71R homozygous mutant was 40% of that of wild-type UGT1A1 and the mRNA levels were not significantly reduced, suggesting that the protein is unstable and easily degradable in the G71R mutation [24]. In addition, Nie et al. collected liver samples from 88 Han Chinese individuals, and UGT1A1 activity was determined by HPLC using bilirubin as a substrate. It has been shown that the G71R mutation reduces mRNA and protein expression levels of UGT1A1 up to 40–60% and reduces enzyme activity [25]. Yamamoto et al. established *in vitro* cell models of the UGT1A1 wild-type and the G71R homozygous and heterozygous mutants using plasmid pcDL and COS-7 cells and reported that the relative activity of the homozygous model of G71R was 32.2% of the normal value, and the heterozygous model of G71R was 60.2% [26]. Wada et al. established *in vitro* cell models of the UGT1A1 wild-type and the G71R homozygous and heterozygous mutants using plasmid pcDNA and COS-7 cells. They found that the UGT1A1 activity of the homozygous and heterozygous models with G71R was 24 and 80% of normal UGT1A1 activity, respectively [20]. In our study, we established *in vitro* cell models of the UGT1A1 wild-type and the G71R homozygous and heterozygous mutants using a lentiviral vector and COS-7 cells. We found that the homozygous and heterozygous models with G71R had 22 and 71% of the normal UGT1A1 activity, respectively, which was similar to that in previous studies. The differences may be related to expression vectors and detection methods. Moreover, this result explained the outcome of a previous study in which a higher incidence of severe hyperbilirubinemia was observed in G71R homozygous individuals than in wild-type and heterozygous individuals [19]. Therefore, G71R mutations led to a decline in enzyme activity, and homozygous enzyme activity decreased more than heterozygous enzyme activity. The reason may be that the G71R mutation affects the spatial conformation of the protein, reducing the binding of the enzyme to the substrate. However, whether the G71R mutation affects the expression of mRNA and protein is still unclear and requires further investigation.

Some UGT1A1 enzyme activity can still be observed in patients with GS and CN-II. Currently, phenobarbital is widely used in clinical situations to induce UGT1A1 enzyme activity. UGT1A1 enzyme activity is completely absent in patients with CN-I, and the clinical treatment for these patients is liver transplantation. With the development of modern molecular biology and gene cloning technologies, gene therapy has great potential for treating severe hyperbilirubinemia due to a severe deficiency or complete loss of UGT1A1 enzyme activity. At present, some studies have established *in vitro UGT1A1* expression models using pCR3.1 [27,28], pcDNA3.1 (+/−) [29], pcDL [30], adenovirus [31], and so on; however, the use of a lentivirus system for this purpose has not been reported. Lentivirus vectors have a wide spectrum of infected cells and high infection efficiency. These viruses can accommodate large fragments of foreign target genes and integrate their DNA into the cell genome, ensuring that its expression is stable and efficient for a long time *in vivo*. In addition, lentiviruses are safe in the long term and have good tolerance in gene therapy [32]. Therefore, a lentivirus vector was used in this study. Ronzitti et al. reported that using the adeno-associated virus (AAV) vector expressing the *UGT1A1* transgene led to the CN-I phenotype in Gunn rats being rescued and long-term (>1 year) correction of the disease [33]. Bočkor et al. showed that repeated AAV-mediated gene transfer by serotype switching led to long-lasting therapeutic levels of the UGT1A1 enzyme in a CN-I mouse model [34]. In this study, a lentiviral vector expressing *UGT1A1* was successfully constructed by genetic engineering, providing the foundation for the next step in animal studies.

The generated conjugated bilirubin could not be directly quantified because unconjugated and conjugated bilirubin have unstable chemical properties and a standard product for conjugated bilirubin is lacking. To further improve the accuracy of the experiment, it was necessary to overcome the limitation of unstable conjugated bilirubin; therefore, attempts were made to synthesize stable standard conjugated bilirubin or find substitutes. The *K*_*m*_ value of enzymes is a direct indicator that reflects the affinity of the reaction enzyme to the substrate. To further demonstrate the role of the G71R mutation in the catalytic function of UGT1A1, the difference in the *K*_*m*_ value between the G71R mutant and wild-type UGT1A1 needs to be studied.

In conclusion, we successfully established *in vitro* cell models of the UGT1A1 wild-type and the G71R homozygous mutant and the heterozygous mutant using a lentiviral vector. Furthermore, the catalytic activity for unconjugated bilirubin was lower in the mutant G71R enzyme than in the UGT1A1 wild-type enzyme, and a weaker effect was observed in the homozygote. The use of our model may provide an ideal means to reveal the pathogenic mechanism of the G71R mutation at the molecular level, providing a basis for the clinical diagnosis, prevention, and treatment of unconjugated neonatal hyperbilirubinemia.
